# Novel ST3871 *Escherichia coli* exhibits diverse transmission modes, carrying the *tet*(X4) resistance gene

**DOI:** 10.1128/spectrum.00521-25

**Published:** 2026-01-23

**Authors:** Qing Wang, Chengye Wang, Muhammad Shoaib, Xiaohui Yao, Xi Chen, Yanhua Qiu, Guonian Dai, Honglin Lin, Weiwei Wang, Jiyu Zhang

**Affiliations:** 1 Chinese Academy of Agricultural Sciences, Lanzhou Institute of Husbandry and Pharmaceutical Sciences 12661, Lanzhou, Gansu Province, People's Republic of China; 2 College of Veterinary Medicine, Gansu Agricultural University 74661, Lanzhou, Gansu Province, People's Republic of China; 3 Key Laboratory of New Animal Drug Project of Gansu Province, Lanzhou, Gansu Province, People's Republic of China; 4 Ministry of Agriculture and Rural Affairs, Key Laboratory of Veterinary Pharmaceutical Development, Lanzhou, Gansu Province, People's Republic of China; 5 Shanghai Veterinary Research Institute, Chinese Academy of Agricultural Science 118161 , Shanghai, People's Republic of China; Universidad Nacional Autonoma de Mexico - Campus Morelos, Cuernavaca, Mexico

**Keywords:** *Escherichia coli*, antimicrobial resistance, tigecycline resistance, *tet*(X)

## Abstract

Although tigecycline is strictly restricted for use in animals, plasmid-mediated *tet*(X) is increasingly being detected in swine and poultry production, which may be associated with the highly intensive farming model and the use of antibiotics as feed additives. *Escherichia coli*, as one of the important reservoirs of *tet*(X), has attracted worldwide attention due to the complex plasmid background and the diversified mobile genetic elements (MGEs). This study isolated nine *tet*(X)-positive *E. coli* belonging to a novel sequence type (ST), ST3871, from a commercial swine farm in Hebei, China. Phylogenetic analysis and pulsed-field gel electrophoresis (PFGE) demonstrated that clonal transmission drove the farm-level prevalence of these strains. The ST3871 *tet*(X)-positive *E. coli* exhibited resistance to multiple antibiotics and carried various resistance genes. Whole-genome sequencing (WGS) and southern blotting confirmed that these strains carried the plasmid-mediated *tet*(X4) gene. Conjugation assays revealed that *tet*(X4)-positive plasmids could be transferred into *E. coli* and *Salmonella*. Although the limited sampling range of this study limits further epidemic analysis, the emergence of *tet*(X)-mediated tigecycline resistance in a novel ST of *E. coli* undeniably confirms that the *tet*(X) gene has resided in a wide variety of ST clones, highlighting the significant contribution of *E. coli* to the dissemination of *tet*(X).

**IMPORTANCE:**

*E. coli* carrying *tet*(X) has spread globally, with the most extensive distribution observed in Asia. This study revealed the prevalence of a novel ST3871 *E. coli* carrying the plasmid-mediated *tet*(X4) gene on a commercial swine farm in Hebei, China, indicating that the *tet*(X) gene, particularly plasmid-mediated *tet*(X), has been distributed across a wide variety of *E. coli* ST clones. This undoubtedly poses a threat to public health, necessitating comprehensive strategies and continuous monitoring to control it.

## INTRODUCTION

Antimicrobial resistance (AMR) has emerged as one of the paramount global health challenges critically jeopardizing therapeutic efficacy in human medicine and undermining food security (1). A prominent manifestation of this crisis involves the alarming global proliferation of high-risk resistant genes, such as the flavin-dependent monooxygenase encoded by *tet*(X) (2). For severe infections caused by carbapenem-resistant bacteria (CRB), treatment options are limited (3), and tigecycline is inevitably relied on. However, the emergence of the *tet*(X) gene weakens this key line of defense (2). Particularly, several *tet*(X) variants, such as *tet*(X3) and *tet*(X4), have been reported in transferable plasmids of *Enterobacteriaceae* and *Acinetobacter* from animals and humans (2, 4, 5). Zhang et al. (6) documented 613 *tet*(X)-positive *E. coli* and found these strains are mainly distributed in Asia, particularly in China, Pakistan, Singapore, and Malaysia. In China, tigecycline is only approved for the treatment of human infections, whereas its use in food-producing animals is not authorized. However, *tet*(X) has been found in multiple food-producing animals, particularly swine and poultry (3, 5), suggesting that swine and poultry serve as significant reservoirs of tigecycline resistance.

The increase of antimicrobial resistance in gram-negative bacteria is not only faster than that in gram-positive bacteria, but also there are fewer new and developmental antibiotics against gram-negative bacteria (7, 8). The plasmids and associated mobile genetic elements (MGEs) in gram-negative bacteria can readily spread resistance within bacterial populations (9, 10). *E. coli* is the most common opportunistic pathogen and considered a vital reservoir for antibiotic resistance genes (ARGs) (3, 11–14). *E. coli* carrying *tet*(X) was highly diversified globally, especially in China (6, 15), of which ST10 and ST761 were reported to be the most prevalent STs and identified from various sources and hosts (16). Furthermore, multiple STs of *tet*(X)-positive *E. coli* were widely distributed worldwide or spread rapidly within a limited area (5, 15). Here, we report an epidemic of a novel ST3871 *E. coli* harboring *tet*(X4) in a commercial swine farm in Hebei province, China.

## MATERIALS AND METHODS

### Strain collections, *tet*(X) screening, and bacterial species identification

A total of 245 non-duplicate fecal samples were collected from a commercial pig farm in Hebei province, China. Approximately 10 fecal samples were randomly collected per lairage. All pig fecal samples were collected if the number of pigs in a lairage was less than 10. The samples were transferred into sterile tubes containing 5 mL brain heart infusion (BHI) broth (Huan Kai Microbial, China) using sterile and disposable swabs. All samples were inoculated on MacConkey plates supplemented with 2 μg/mL tigecycline for overnight cultivation. All strains were screened for a conserved region in *tet*(X) using a primer pair described in the previous report (2), with further verification by Sanger sequencing. Three non-duplicate fecal samples (each collected from an independent lairage) were randomly selected and cultured on MacConkey agar without antibiotics to obtain tigecycline-susceptible strains. Bacterial species identification was performed for all strains using matrix-assisted laser desorption/ionization time-of-flight mass spectrometry (MALDI-TOF MS, score values > 2.0, secure identification) (17) and 16S rDNA (sequence similarity > 99%, secure identification) (18).

### Conjugation assay

The transferability of *tet*(X) genes was determined using the filter mating method, sodium azide-resistant *E. coli* J53, and rifampicin-resistant *Salmonella* LGJ2 (donated by the College of Veterinary Medicine, South China Agricultural University) as the recipient. Overnight cultures of strains carrying *tet*(X), recipients J53 and LGJ2, were grown at log phase in brain heart infusion (BHI) broth, respectively. A mixture of donor and recipient strains at a ratio of 1:1 was applied on a 0.22-μm filter membrane following incubation at 37°C for 16 h. The conjugation mixtures were transferred to MacConkey agar plates supplemented with 4 μg/mL tigecycline and 100 μg/mL sodium azide or 200 μg/mL rifampicin. Transconjugants were used for bacterial species identification using 16S rDNA and Sanger sequencing and for *tet*(X) identification using PCR amplification and Sanger sequencing. Furthermore, antimicrobial susceptibility testing was performed for transconjugants against tigecycline. Transfer frequency was calculated based on colony counts of the transconjugant and recipient cells in triplicate, as previously reported (3).

### PFGE (*Xba*I-PFGE and S1-PFGE) and southern blotting

The *Xba*I nuclease (Thermo Scientific, USA) digested PFGE was executed on the CHEF Mapper XA platform (Bio-Rad, USA) using *Salmonella* H9812 as the reference standard. PFGE bands were analyzed on the BioNumerics (v7.6) platform. S1-nuclease pulsed-field gel electrophoresis (S1-PFGE) and southern blotting were performed to determine the location of the *tet*(X) gene as previously described (19). Briefly, *Salmonella* H9812 was used as the molecular quality standard reference strain. The *tet*(X)-positive strains were wrapped in 1% agarose gel (Gold Agarose, Lonza, Switzerland) and digested with 0.5 μL S1 nuclease (Takara, Japan) for 45 min at 37°C. PFGE was carried out under the running conditions of 6 V/cm and a pulse time range of 2.20 s to 54.20 s at 14°C for 19 h using a CHEF Mapper XA System (Bio-Rad, USA). The capillary transfer method was employed to transfer the DNA to a positively charged nylon membrane (Solarbio, China), and the DNA was subsequently immobilized using ultraviolet light at 0.5 J/cm. The hybridization probe was produced using the abovementioned conserved region of *tet*(X). The prepared probe was preheated to 68°C for 10–15 min and hybridized at an appropriate temperature (as calculated according to the kit instructions) for 20 h. The subsequent southern blotting procedures were performed according to the DIG High Prime DNA Labeling and Detection Starter Kit (Roche, Switzerland).

### Antimicrobial susceptibility testing

Minimum inhibitory concentration (MIC) was determined for all *E. coli* strains using broth microdilution in Mueller-Hinton broth (Huan Kai Microbial, China). Antimicrobial susceptibility was tested according to the Clinical and Laboratory Standards Institute (M100-S34) (20), the European Committee on Antimicrobial Susceptibility Testing (EUCAST, version 14.0) (21), and the U.S. Food and Drug Administration (FDA) criteria (https://www.fda.gov/). Eighteen antibiotics (Solarbio, China), including meropenem, aztreonam, ampicillin, ceftazidime, cefepime, gentamicin, chloramphenicol, polymyxin, kanamycin, fosfomycin, ciprofloxacin, sulfamethoxazole, azithromycin, tetracycline, doxycycline, tigecycline, rifampin, and omadacycline, were tested. The breakpoint of tigecycline, omadacycline, and colistin for *E. coli* was interpreted according to the FDA criteria (tigecycline: S = ≤2 μg/mL, I = 4 μg/mL, R = ≥8 μg/mL; omadacycline: S = ≤4 μg/mL, I = 8 μg/mL, R = ≥ 16 μg/mL; colistin: I = ≤2 μg/mL, R = ≥ 4 μg/mL). Meropenem was interpreted according to the European Committee on Antimicrobial Susceptibility Testing breakpoint (S = ≤ 2 μg/mL, R = ≥ 8 μg/mL). The remaining antibiotics panel was interpreted according to the Clinical and Laboratory Standards Institute breakpoints (M100-S34, 2024). *E. coli* ATCC25922 was used as a quality control for antimicrobial susceptibility testing, and the quality control range for ATCC25922 was based on the CLSI criterion.

### Whole-genome sequencing

The DNBSEQ platform (Beijing Genomics Institution, China) was used for whole-genome sequencing. Genomic DNA was randomly sheared to construct three read libraries with lengths of 300–400 bp using physicochemical methods. The paired-end fragment libraries were sequenced. Raw reads of low quality from paired-end sequencing were discarded. The sequenced reads were assembled using SOAPdenovo v.2.0.4. One *tet*(X)-positive *E. coli* strain, W5-C5E1, was selected for ONT long-read sequencing. Briefly, the genome was sequenced using the Oxford Nanopore and DNBSEQ platforms. The corrected ONT long-read reads were generated by hybrid assembly with DNBSEQ short reads.

### Bioinformatics and genetic environment analyses

Sequence typing was identified for each strain using Pathogenwatch (https://pathogen.watch/) (22). Serotype identification and plasmid replicon typing were performed using SerotypeFinder 2.0 and PlasmidFinder 2.1 in the Center for Genomic Epidemiology. Given the clinical importance of ARGs and virulence factors in *E. coli*, each strain was screened for known antibiotic resistance and virulence genes using ResFinder version 4.6.0 and VFanalyzer (>90% identity) (http://www.mgc.ac.cn/VFs/main.htm). To determine whether recombination between two IS*Vsa3* elements could result in the formation of a *tet*(X4)-carrying minicircle, inverse PCR assays were conducted using a primer pair described in the previous report (2).

### Phylogenetic analysis

**Fig 1:**
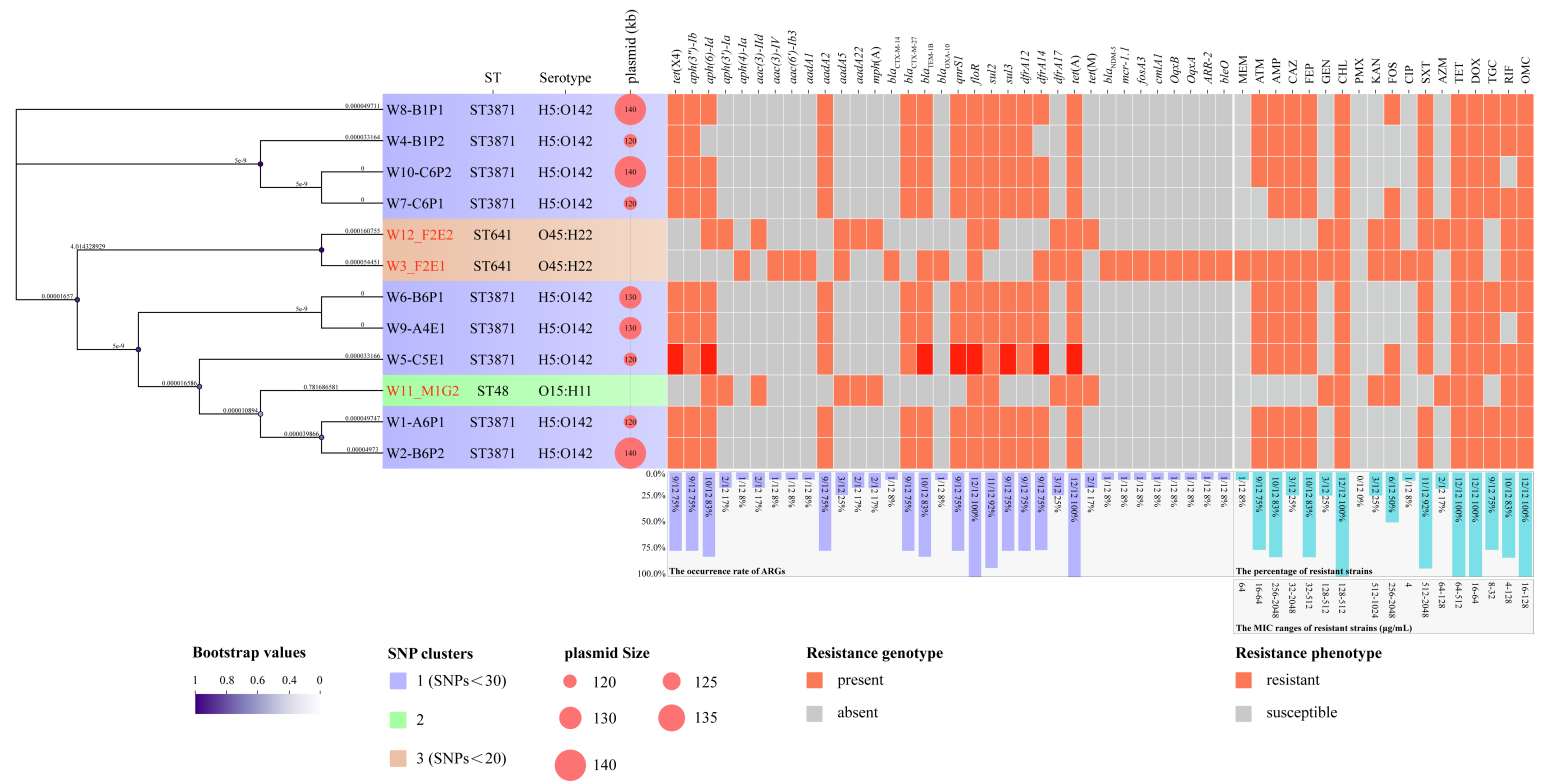
An ML cgSNP-based phylogenetic tree, STs, serotype, sizes of *tet*(X)-positive plasmids, and resistant genotype and phenotype. The phylogenetic tree is built based on the strains of this study. All strains are grouped into three SNP clusters based on their cgSNP. Leaf names labeled in red designate *tet*(X)-negative strains, and names in black indicate *tet*(X)-positive strains. Bootstrap values are shown as circles. Branch lengths are displayed as numbers on each branch of this tree.

A maximum-likelihood (ML) linear phylogenetic tree based on core-genome single-nucleotide polymorphisms (cgSNPs) was constructed using the *E. coli* strains of this study (Fig. 1). An ML circular phylogenetic tree based on cgSNP was built using the 95 *tet*(X)-positive *E. coli* strains containing 61 ST types retrieved from the National Center for Biotechnology Information (NCBI) database and the *E. coli* strains of this study (Fig. 3). CgSNP was extracted using Snippy in the Harvest package with a default parameter. Genome W5 was used as the reference genome. Each genome in the linear phylogenetic tree produced a core genome of approximately 60 kb, and each genome in the circular phylogenetic tree produced a core genome of approximately 170 kb. The ML phylogenetic trees were built with FastTree (FastTree 2.2) and further visualized in iTOL v7 using the corresponding features for each strain.

## RESULTS

Nine *tet*(X)-positive *E. coli* strains were identified from 245 pig fecal samples with an isolation rate of 3.67% (a 95% confidence interval [CI] of 1.9 to 6.8%) from a commercial pig farm in Hebei province, China. Five of the nine strains were from two lairages (lairage 1, *n* = 3; lairage 2, *n* = 2), and the rest were from four individual lairages. Three *tet*(X)-negative strains were from three individual lairages. All strains (*n* = 12) were verified as *E. coli* using MALDI-TOF MS and 16S rDNA.

### Antimicrobial resistance phenotype

Antimicrobial susceptibility testing was performed using a broth microdilution assay, and minimum inhibitory concentrations (MICs) were calculated. It showed that all *tet*(X)-carrying *E. coli* strains were resistant to tetracycline (256–512 μg/mL), doxycycline (32–64 μg/mL), tigecycline (8–32 μg/mL), omadacycline (32–128 μg/mL), ampicillin (256–2,048 μg/mL), ceftazidime (16–64 μg/mL), cefepime (32–512 μg/mL), chloramphenicol (128–256 μg/mL), and sulfamethoxazole (512–2,048 μg/mL), but exhibited susceptibility to meropenem, polymyxin, kanamycin, gentamicin, ciprofloxacin, and azithromycin (Fig. 1 and Supplementary Data). Most of the *tet*(X)-positive *E. coli* strains showed resistance to aztreonam (*n* = 8, 89%), rifampin (*n* = 7, 78%), and fosfomycin (*n* = 3, 33%). Moreover, three *tet*(X4)-negative *E. coli* strains also showed resistance to omadacycline, gentamicin, chloramphenicol, kanamycin, fosfomycin, tetracycline, doxycycline, and rifampin (Fig. 1 and Supplementary Data).

### Bioinformatics analysis

The assembled draft genomes of all strains yielded an average total genome size of 5.04 MB, with a GC content of 50.3–50.7%. Serotype analysis revealed that all *tet*(X)-positive *E. coli* belonged to O142: H5, and *tet*(X)-negative *E. coli* strains belonged to O45: H21 and O15: H1. Three STs were identified, including ST3871 (*n* = 9), ST641 (*n* = 2), and ST48 (*n* = 1). Notably, all *tet*(X)-positive strains were identified as ST3871, and no other variants of the *tet*(X) family were identified, except for *tet*(X4) (Fig. 1 and Supplementary Data 1). Apart from *tet*(X), a median of 12 AMR (range 11–13) genes was detected from the assembled genome of each *tet*(X)-positive strain. These genes mainly encoded resistance to aminoglycoside (*aph(3")-Ib*, 9/9; *aadA2*, 9/9; *aph(6)-Id*, 7/9), monobactam (*bla*_TEM-1B_, 9/9), phenicol (*floR*, 9/9), fluoroquinolone antibiotic (*QnrS1*, 9/9), sulfonamide (*sul2*, 9/9; *sul3*, 9/9), tetracycline (*tet*(A), 9/9), carbapenem (*bla*_CTX-M-27_, 9/9), and diaminopyrimidine (*dfrA12*, 9/9; *dfrA14*, 7/9) (Fig. 1 and Supplementary Data).

VFanalyzer revealed that all *tet*(X)-positive strains carried more than 20 virulence factors with a similar virulence factor spectrum. These virulence genes mainly encoded adherence proteins (afimbrial adhesin AFA-I: *afaB* and *afaC*; *E. coli* common pilus [ECP]: *ecpA-E; E. coli* laminin-binding fimbriae [ELF]: *elfC*, *elfD*, and *elfG*; hemorrhagic *E. coli* pilus [HCP]: *hcpA-C*; *P* fimbriae: *papI*; type I fimbriae: *fimD*, *fimF*, *fimG*, and *fimH*), autotransporter (*aatA*), invasion proteins (invasion of brain endothelial cells: *ibeB* and C; *tia*), Non-LEE encoded TTSS effectors (*espL1*, *espL4*, *espR1*, *espX1*, *espX4*, and *espX5*) and toxin proteins (hemolysin/cytolysin A: *hlyE*/*clyA*) (Supplementary Data).

### Phylogenetic analysis based on the strains of this study

**Fig 2:**
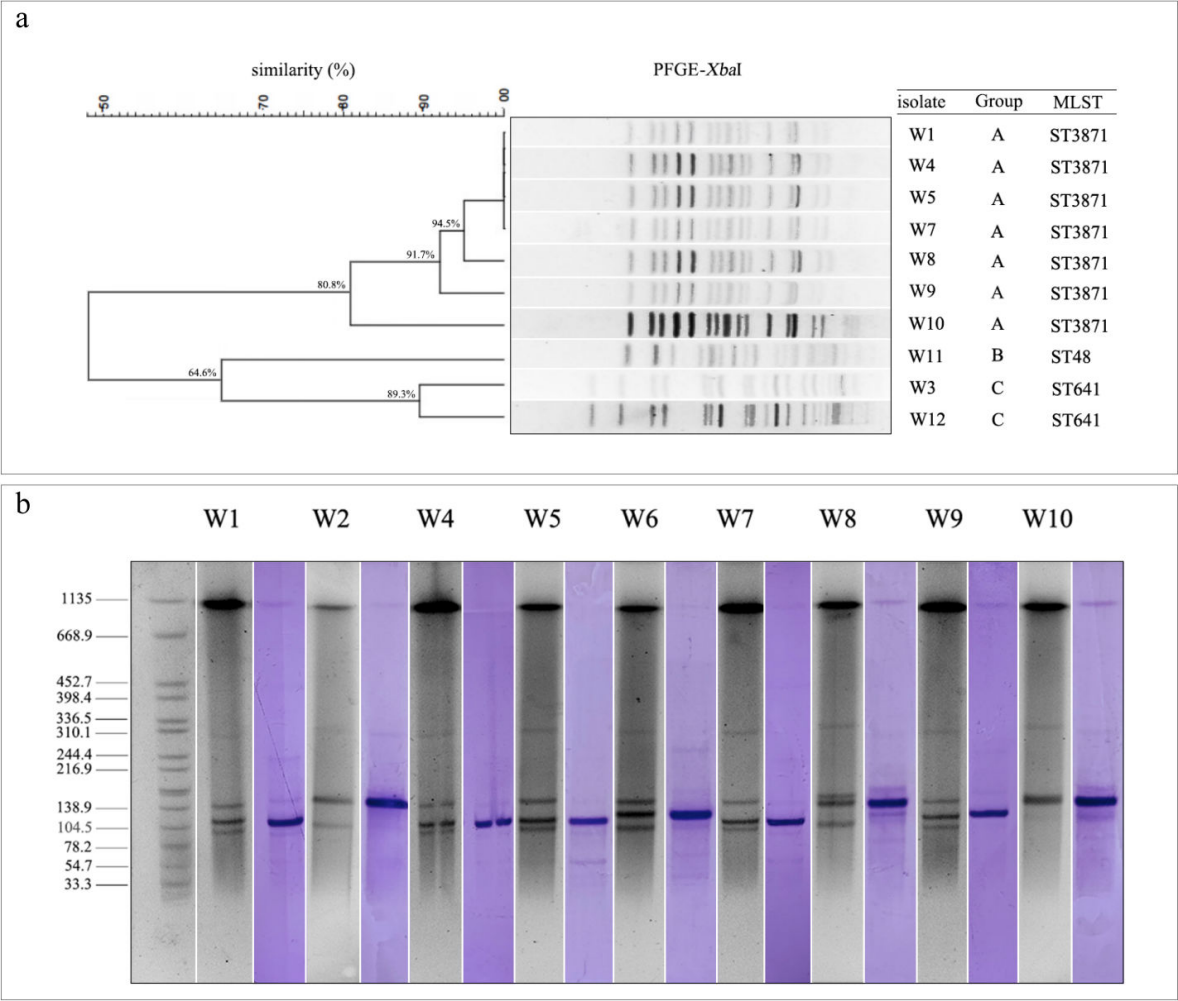
(**a**) *Xba*I-digested PFGE dendrogram. (**b**) *tet*(X)-positive vehicle location using S1-PFGE and southern blotting. PFGE grouping is based on electrophoretic band similarity (≥80%).

To assess the genetic similarity, all strains were subjected to the *Xba*I-PFGE analysis. Even after numerous attempts, the stripes of two *tet*(X)-positive *E. coli* strains still generated diffusion, so their results were excluded (Fig. S1 shows unclear bands). The remaining strains were clustered into three PFGE clusters based on ≥80% electrophoretic band similarity, including seven *tet*(X)-positive strains in cluster A, one *tet*(X)-negative strain in cluster B, and two *tet*(X)-negative strains in cluster C (Fig. 2a).

An ML-cgSNP phylogenetic tree was built based on the strains of this study. All strains were grouped into three SNP clusters, including nine *tet*(X)-positive strains in cluster 1 (purple), one *tet*(X)-negative strain in cluster 2 (green), and two *tet*(X)-negative strains in cluster 3 (yellow) (Fig. 1). The strains in each cluster showed high genetic similarity, which was reflected by low-level core genome diversity. The SNP range of cluster 1 (purple) was 0–30 (Fig. 1). The SNP range of cluster 3 (green) was 0–20 (Fig. 1). In the current study, PFGE clusters were consistent with ST types and similar to the SNP clusters.

### Vehicle location of *tet*(X) and transferability for *tet*(X)-positive plasmids

S1-PFGE and southern blotting were performed to locate the vehicles for *tet*(X). All *tet*(X)-positive *E. coli* strains carried three to five plasmids, with the sizes of 78 to 310 kb (Fig. 2b and Supplementary Data). The *tet*(X) was confirmed to be carried by the plasmids with the sizes of ≈120–150 kb (Fig. 2b and Supplementary Data).

The filter-mating conjugation assay was conducted to evaluate the horizontal transfer potential of *tet*(X)-positive plasmids. Results indicated that the *tet*(X) gene could be successfully transferred to *E. coli* J53 and *Salmonella* LGJ2 at frequencies of 1.0 × 10^−2^ to 9.4 × 10^−2^ and 9.4 × 10^−7^ to 5.1 × 10^−6^ cells per donor cell, respectively. The transconjugants exhibited a twofold change in the tigecycline MIC, reflected in either a twofold increase or a twofold decrease, as determined by antimicrobial susceptibility testing (Supplementary Data). S1-PFGE and southern blotting confirmed that the *tet*(X)-positive plasmids carried by donor strains could be classified into three types (≈120 kb: W1, W4, W5, and W7; ≈130 kb: W6 and W9; ≈150 kb: W2, W8, and W10) based on their sizes. Thus, the transconjugants (*E. coli* J53 and *Salmonella* LGJ2) of one donor strain from each type (W2, W6, and W7) were selected for S1-PFGE and southern blotting. The analysis revealed that only the *tet*(X)-positive plasmid was transferred into the recipient strains. The sizes of *tet*(X)-positive plasmids in the recipient strains (the transconjugants of strains W2, W6, and W7) were similar to those in the donor strains (Fig. S2).

### Phylogenetic analysis based on the public database

**Fig 3:**
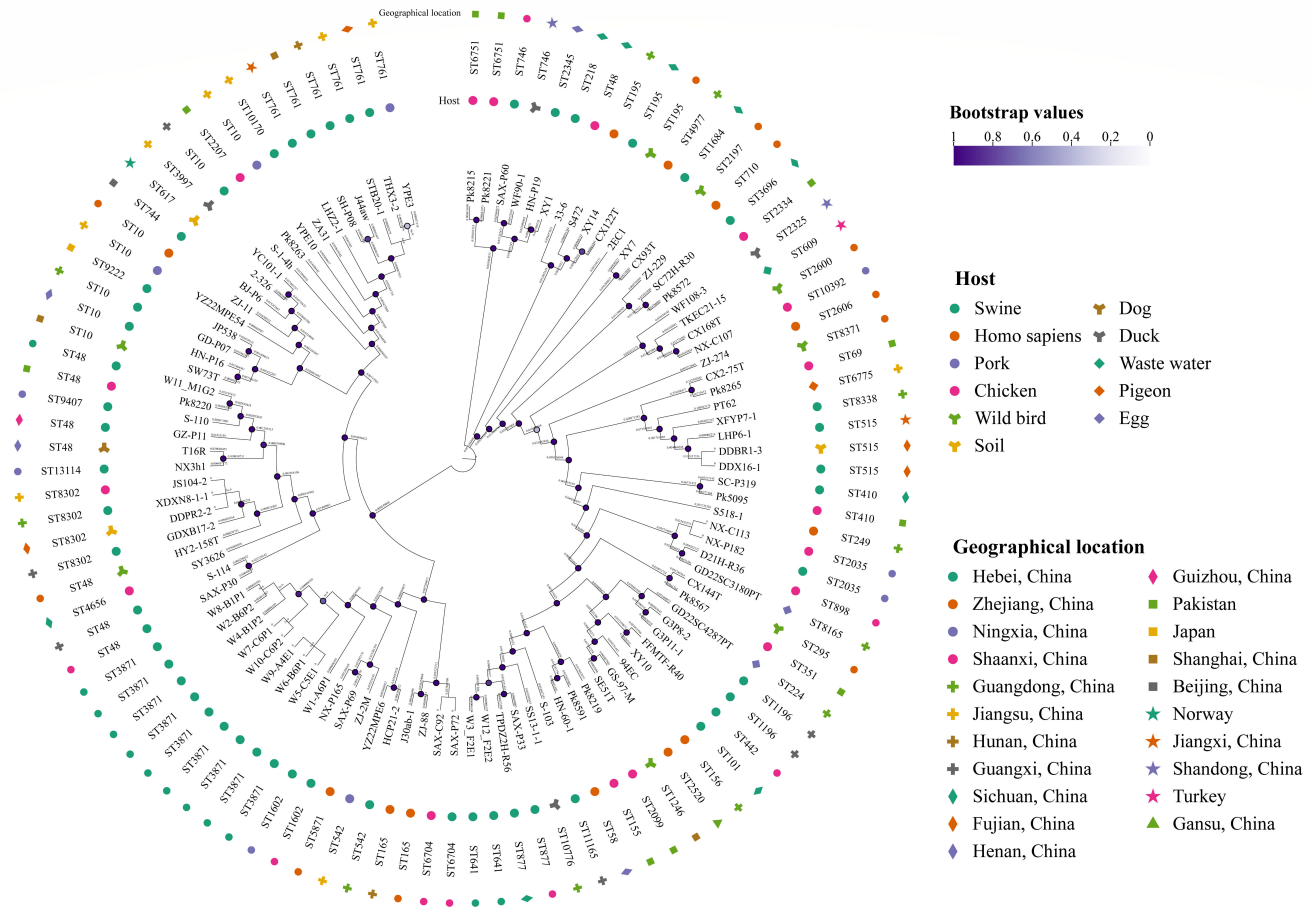
An ML cgSNP-based phylogenetic tree is built using the *E. coli* strains from the current study and the NCBI database. Bootstrap values are shown as circles. Branch lengths are displayed as numbers on each branch of this tree.

To further analyze the genetic similarity of ST3871 *E. coli*, we retrieved the EnteroBase database and identified four ST3871 *E. coli* strains. These strains were typed using housekeeping genes or cgMLST, and their genomes could not be retrieved via the BioProject number. Given this, 93 *tet*(X)-positive *E. coli* genomes (including 61 ST types) were selected from NCBI databases. These genomes were sourced from 19 regions or countries, including Pakistan, Turkey, Norway, Japan, and 16 provinces or municipalities of China. Subsequently, these genomes and those from this study were used to build a cgSNP-ML phylogenetic tree (Fig. 3). Results showed that no strain exhibited genetic similarity to ST3871 *tet*(X)-positive *E. coli*, as reflected in high-level core genome diversity (>10,000 SNPs).

### Plasmid analysis

Based on the comprehensive data analysis, one *tet*(X)-positive strain (W5) was selected for Oxford Nanopore Technologies (ONT) long-read sequencing to better understand the genetic context of plasmid-mediated *tet*(X). This analysis revealed that the strain carried three plasmids, and *tet*(X4) was located in plasmid-1 (pW5-C5E1) with a size of 121,928 bp. This plasmid was typed as a multireplicon plasmid, IncFIA-IncX1, and predicted to be conjugative. *Mpf* (mating pair formation, typed as *mpf*_F), *oriT*, and relaxase (typed as MOBF) could be identified in this plasmid. In addition to *tet*(X4), seven resistance genes, including *aph(6)-Id*, *bla*_TEM-1B_, *qnrS1*, *floR*, *sul3*, *drfA14*, and *tet*(M), were located in this plasmid (crimson cells, Fig. 1). The resistant genes carried by *tet*(X4)-positive plasmid accounted for 61.54% of the resistant genes carried by strain genome, reflecting the fact of co-selection in driving plasmids’ persistence. Plasmid-2 was also typed as a multireplicon plasmid, IncFIB-IncH1B, and predicted to be mobilizable. Relaxase (MOBH) could be identified, but *mpf* and *oriT* could not be identified in this plasmid. Plasmid-3 was typed as IncI and predicted to be conjugative. *Mpf* (typed as *mpf*_I), relaxase (typed as MOBP), and *oriT* could be identified in this plasmid. No resistance genes were identified in plasmid-2 and -3.

### Genetic context of *tet*(X4)

**Fig 4:**
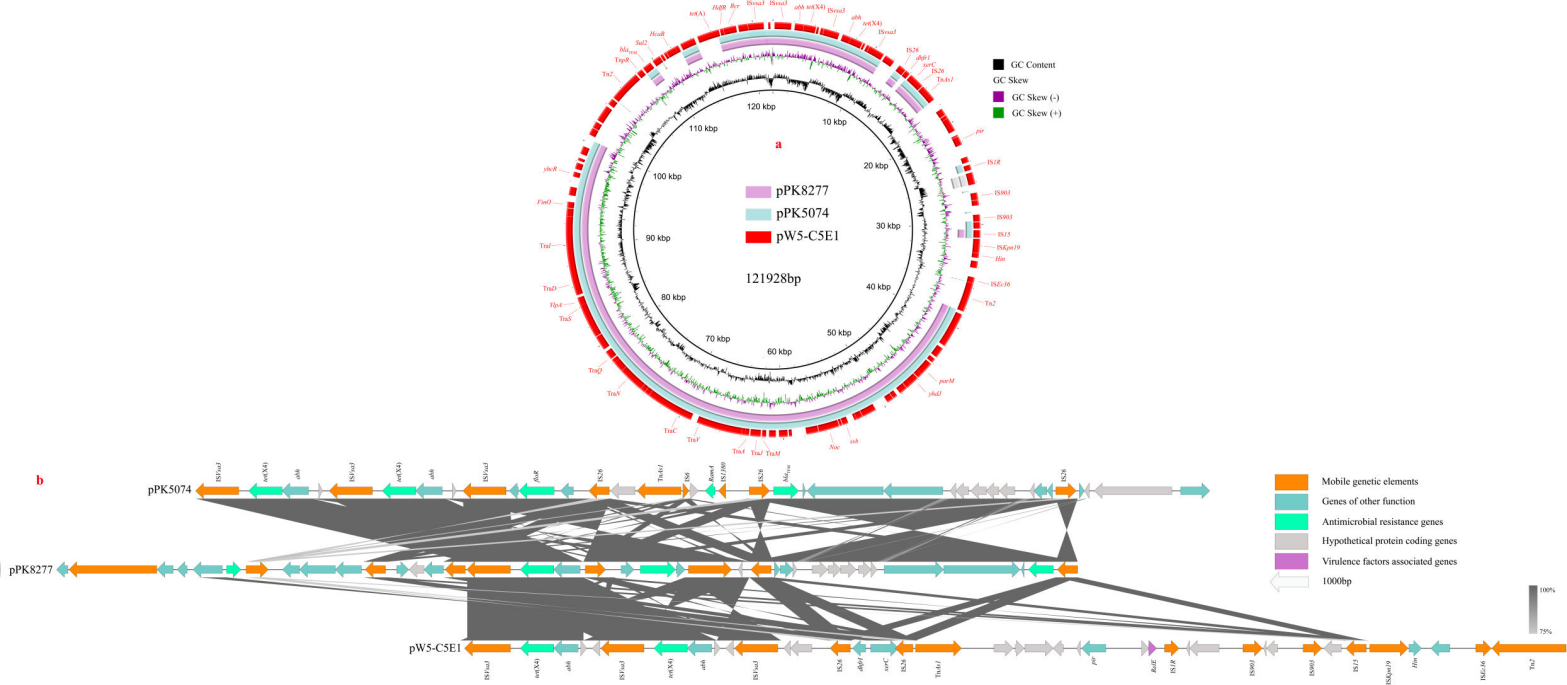
Circular and linear comparisons between the *tet*(X)-positive plasmid of this study and the similar plasmids of the public database. (**a**) A circular comparison is based on the whole plasmid genomes of pPK8277, pPK5074, and pW5-C5E1. (**b**) A linear comparison is constructed for the region of about 35,000 bp.

Two closely linked *tet*(X4) genetic contexts were located within a region of 10,805 bp, with the gene arrangement, IS*Vsa3*-ORF2-*abh-tet*(X4)-IS*Vsa3*-ORF2-*abh-tet*(X4)-IS*Vsa3* (Fig. 4). The genes in this region encoded flavin-dependent monooxygenase (*tet*(X4)), IS*91*-like family transposase (IS*Vsa3*), alpha/beta hydrolase (*abh*), and hypothetical protein, respectively. IS*Vsa3* is a vital horizontal gene transfer element in the spreading of *tet*(X), and its three copies in the same direction and complete size were identified in this region (Fig. 4). BLASTn analysis confirmed that the region, IS*Vsa3*-ORF2-*abh-tet*(X4)-IS*Vsa3*, was almost identical (100% coverage and 99.95% identity; one base mismatch and two bases’ gap) to the IS*Vsa3*-mediated minicircle carrying *tet*(X4) in *E. coli* plasmid p47EC (MK134376, first reported to carry the IS*Vsa3*-mediated *tet*(X4) minicircle) (2). Subsequently, inverse PCR was used to examine whether the region was circular. Sequence analysis revealed a region that consisted of the *tet*(X4)-carrying central region and one copy of IS*Vsa3*.

The BLASTn analysis revealed that pW5-C5E1 exhibited 99.28% identity and 71% coverage with pPK8277 (CP080134.1) in a chicken-derived *E. coli* PK8277 and 99.16% identity and 72% coverage with pPK5074 (CP072807.1) in a human-derived *E. coli* PK5074. These plasmid genomes were sourced from Faisalabad, Pakistan. Circular comparison of the complete plasmid genomes revealed two highly similar regions with the sizes of 60 and 13 kb, of which the genes in the region of 13 kb encoded *tet*(X4) genetic context (Fig. 4a). The linear comparison of the 35 kb regions containing *tet*(X) revealed that pPK5074 carried two closely linked IS*Vsa3-*mediated *tet*(X4) genetic contexts, but pPK8277 carried a single IS*Vsa3-*mediated *tet*(X4) genetic context (Fig. 4B). These regions mostly differed in the upstream and downstream of the *tet*(X4) genetic context, where they contained the genes encoding mobile elements and antimicrobial resistance (Fig. 4b).

**Fig 5:**
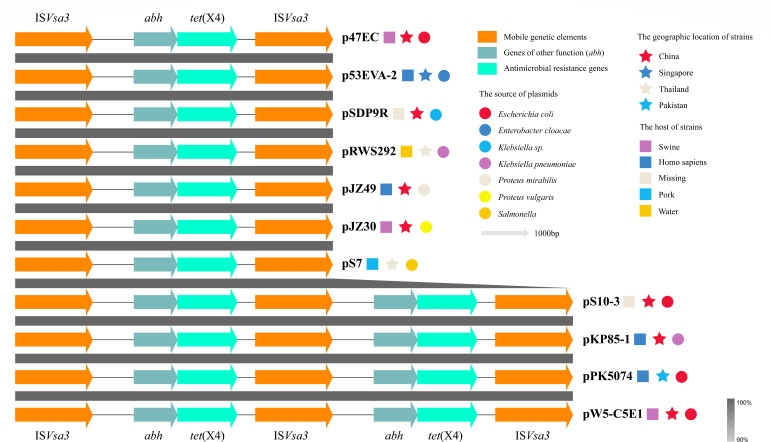
Linear comparison of the *tet*(X) genetic context between pW5-C5E1 and the plasmids carried by various bacteria of the public database. The similarity among *tet*(X) genetic contexts was identified at 90 to 100%.

Further BLASTn analysis revealed the IS*Vsa3-*mediated *tet*(X4) genetic context could be identified in the plasmids of various bacterial hosts retrieved from the NCBI database. Thus, 10 plasmid-mediated *tet*(X4) genetic contexts and the *tet*(X4) genetic context in pW5-C5E1 were used to construct a linear comparison. Analysis found that seven plasmids carried a single IS*Vsa3-*mediated *tet*(X4) genetic context, and four plasmids carried two closely linked IS*Vsa3-*mediated *tet*(X4) genetic contexts (Fig. 5). Furthermore, these plasmids were carried by multiple bacterial hosts, including *E. coli*, *Enterobacter cloacae*, *Klebsiella*, *Proteus*, and *Salmonella*, but their IS*Vsa3-*mediated *tet*(X4) genetic context displayed high similarity (100% coverage and >99.5% identity) (Fig. 5).

## DISCUSSION

The current study revealed the epidemic of a novel ST3871 *E. coli* carrying *tet*(X) in a commercial swine farm in Hebei province, China. PFGE and phylogenetic analysis revealed the presence of high genetic similarity among these strains. Meanwhile, these strains also exhibited similar resistance gene backgrounds. This indicates that clonal transmission drove the prevalence of the ST3871 *E. coli* carrying *tet*(X) in this swine farm. Nine *tet*(X)-positive ST3871 *E. coli* were isolated from 245 samples with an isolation rate of 3.67%. This isolation rate is higher than that of another swine farm in Hebei province (5). However, previous studies (3, 5) found that *tet*(X)-positive *E. coli* exhibit a sporadic distribution in China, reflected in considerable differences in isolation rates across provinces or farms within the same province. Therefore, we consider that the 3.67% isolation rate can be used only to define the farm under this investigation. Short-read sequencing demonstrated that all *E. coli* isolates carried *tet*(X4). Since the initial detection in China in 2018 (2), *tet*(X4) has rapidly spread nationwide and been detected in dozens of provinces, including Liaoning, Hebei, Henan, Shanxi, Jiangsu, Zhejiang, Shaanxi, Gansu, Sichuan, Hunan, and Hubei (3, 5). In addition to China, this gene has been widely distributed worldwide, including Canada, Thailand, the Republic of Korea, Iran, South Africa, Turkey, Iraq, the United Kingdom, Pakistan, Norway, and Singapore (23). Compared to the earlier-detected *tet*(X) and *tet*(X2), *tet*(X4) exhibits a more potent tigecycline resistance phenotype (24). To date, *tet*(X4) has become the most widely distributed variant in the *tet*(X) family (3, 5) and is continuously detected in various bacteria like *Acinetobacter*, *Escherichia*, *Proteus*, *Raoultella*, *Klebsiella*, *Citrobacter*, and *Enterobacter* (24). Among these, *E. coli* is considered one of the most important reservoirs for *tet*(X4). Human-, animal-, and environment-derived *tet*(X4)-positive *E. coli* have been reported in hospitals, farms, slaughterhouses, and supermarkets (3, 5, 25–28). The emergence of a novel *E. coli* sequence type, ST3871, carrying *tet*(X4), undeniably confirms that this gene has resided in a wide variety of ST clones and further highlights that *E. coli* plays a crucial role in transmitting this gene. Furthermore, although reports have demonstrated genetic similarity between animal-derived *tet*(X4)-positive *E. coli* and human-derived *E. coli* (5), no substantial evidence exists to support the transmission of the *tet*(X) gene to humans through meat products. Nevertheless, the widespread distribution of the *tet*(X) gene, particularly *tet*(X4), in human and animal populations is an established fact that inevitably poses significant risks to human food safety and public health.

Mobile antimicrobial resistance mechanisms, including *tet*(X), *mcr*, and *bla*_NDM_, represent a plasmid-mediated antibiotic crisis. This study demonstrates that *tet*(X4) was carried by plasmids with sizes of ≈120–150 kb, and the *tet*(X4)-positive plasmid of strain W5 was confirmed as a multireplicon plasmid, IncFIA-IncX1. Previous studies (29) found that double-replicon plasmids, even three-replicon plasmids, could carry *tet*(X4). The presence of multiple replicons in the *tet*(X4)-positive plasmid indicates that plasmid fusion facilitates the spread of *tet*(X4), potentially preventing the loss due to plasmid incompatibility (5), which promotes interactions among plasmids to adapt to a broader range of bacterial hosts. Furthermore, the complexity of *tet*(X4)-carrying plasmids may stem from selection pressures arising from the wide usage of “older” generations of tetracyclines (24). Conjugation assay confirmed that the *tet*(X4)-positive multireplicon plasmid could be transferred into *E. coli* and *Salmonella*, suggesting this plasmid is transferable across bacterial genera. Genomic analysis confirms that this plasmid carries multiple crucial elements, including *oriT*, relaxase, and *mpf*, which play roles in origin, cleavage, and transfer during its horizontal transfer. This plasmid carries the *tra* family, including *traA*, *traC*, *traD*, and *traI*, and the proteins encoded by these genes can recognize and cleave the plasmid’s transfer origin site (*oriT*), thereby initiating single-stranded DNA transfer to facilitate horizontal gene transfer. The *tra* genes also encoded sex pili, which are the structural basis for plasmid conjugation. Furthermore, this plasmid carries Tn*2*, a transposon capable of mobilizing within bacterial genomes. It could carry associated genes to new genomic locations, facilitating their dissemination in the bacterial populations. The emergence of *tet*(X4) in a multireplicon plasmid carrying diverse gene transfer elements may further accelerate its spread into various ecological niches, even into clinically high-risk pathogens.

Similar regions to IS*Vsa3* (≥99% nucleotide sequence identity) have been identified in plasmids or chromosomes of more than 26 bacterial species worldwide (2, 30). Plasmids or chromosomes in various bacterial hosts have been observed to harbor highly similar IS*Vsa3*-mediated *tet*(X4) genetic contexts (2, 5, 24, 29). These suggest that the dissemination of the *tet*(X4) gene critically depends on its flanking insertion sequence, IS*Vsa3*. This element can mobilize its adjacent gene regions via a rolling-circle transposition mechanism (29, 30). Most IS elements typically require two copies (one of which must be intact) to complete transposition, such as the transposition of *mcr-1* mediated by IS*Apl1* (31), whereas IS*Vsa3* can achieve transposition of adjacent DNA sequences using a single copy (29, 30). IS*Vsa3*-mediated *tet*(X4) genetic context is frequently associated with various Inc plasmids, such as IncX1, IncFIA, IncHI1A, IncHI1B, IncR, IncN, IncHI1A-IncR, IncX1-IncN, IncX1-IncR, etc. (29). This study found that the *tet*(X4)-positive plasmid carries a region containing two closely linked IS*Vsa3*-mediated *tet*(X4) genetic contexts, and multiple complete copies of IS*Vsa3* can be identified in this region (Fig. 5). This structure suggests that this plasmid may experience the integration of exogenous IS*Vsa3*-mediated *tet*(X4) minicircle. In addition to the IS*Vsa3* located in the *tet*(X4) genetic context, other IS*Vsa3* can be located in this plasmid, but no IS*Vsa3*-mediated *tet*(X4) circular intermediate is integrated into these sites. This implies that the selection tendency exists in the integration process.

### Conclusion

Due to limited selection, the study did not further expand the sampling scope or extend sampling targets to humans. However, the discovery of *tet*(X4)-positive ST3871 *E. coli* confirms that *tet*(X) has diffused to rare STs of *E. coli*, demonstrating its potent dissemination capability. This study underscores the necessity for sustained genetic surveillance and risk assessment grounded in One Health.

## Data Availability

Genomes of *E. coli* have been deposited in NCBI under BioProject accession no. PRJNA1176315 (the draft genome of *tet*(X)-positive *E. coli*), PRJNA1170472 (the draft genome of *tet*(X)-negative *E. coli*), and PRJNA1178352 (the assembled genome of *tet*(X)-positive *E. coli*, W5-C5E1, using ONT long-read sequencing).

## SUPPLEMENTAL MATERIAL

Supplemental material spectrum.00521-25-s0001.pdf Bioinformatics analysis and MIC.

ASM does not own the copyrights to Supplemental Material that may be linked to, or accessed through, an article. The authors have granted ASM a non-exclusive, world-wide license to publish the Supplemental Material files. Please contact the corresponding author directly for reuse.
