# Supplementary material for: Novel ST3871 Escherichia coli exhibits diverse transmission modes, carrying the tet(X4) resistance gene
Source: Microbiol Spectr. 2026 Jan 23;14(3):e00521-25. doi: 10.1128/spectrum.00521-25 (PMC12955495; doi:10.1128/spectrum.00521-25)
Supplement: Supplemental material — Bioinformatics analysis and MIC. [file spectrum.00521-25-s0001.pdf]

Bioinformatics analysis																	
Short-read sequencing													S1-PFGE and Southern blotting			Long-read sequencing	
	Strain name	Organism name	Taxonomy ID	Genome length	No. contigs	N50	GC content	MLST	Serotype	AMR	Plasmid Inc types	virulence factors(VFanalyzer)	No. plasmids	the size of plasmids	the size of tet(X)-positive plasmids (kb)	Genome length	Inc type
Positive	W1-A6P1	Escherichia coli	562	4927596	149	102987	50.70%	3871	H5 O142	aph(3")-Ib aadA2 aph(6)-Id blaCTX-M-27 blaTEM-1B floR qnrS1 sul2 sul3 tet(X4) tet(A) dfrA12 dfrA14	IncFII, IncI1-I(Alpha), IncFIB(K), p0111, IncX1	afaB afaC ecpA-E elfC elfD elfG hcpA-C papI fimD fimF fimG fimH aatA ibeB ibeC tia espL1 espL4 espR1 espX1 espX4 espX5 hlyE/clyA	4	310-104kb	~120		
	W2-B6P2	Escherichia coli	562	4927132	147	100638	50.70%	3871	H5 O142	aph(3")-Ib aadA2 aph(6)-Id blaCTX-M-27 blaTEM-1B floR qnrS1 sul2 sul3 tet(X4) tet(A) dfrA12 dfrA14	IncFII, IncI1-I(Alpha), IncFIB(K), p0111, IncX1	afaB afaC ecpA-E elfA elfC elfD elfG hcpA-C papI fimD fimF fimG fimH aatA ibeB ibeC tia espL1 espL4 espR1 espX1 espX4 espX5 hlyE/clyA	4	310-104kb	~150		
	W4-B1P2	Escherichia coli	562	4922667	122	138150	50.70%	3871	H5 O142	aph(3")-Ib aadA2 blaCTX-M-27 blaTEM-1B floR qnrS1 sul2 sul3 tet(X4) tet(A) dfrA12	IncFII, IncI1-I(Alpha), IncFIB(K), p0111, IncX1	afaB afaC ecpA-E elfC elfD elfG hcpA-C papI fimD fimF fimG fimH ibeB ibeC tia espL1 espL4 espR1 espX1 espX4 espX5 hlyE/clyA	4	310-104kb	~120		
	W5-C5E1	Escherichia coli	562	4948720	122	138454	50.70%	3871	H5 O142	aph(3")-Ib aadA2 blaCTX-M-27 blaTEM-1B floR qnrS1 sul2 sul3 tet(X4) tet(A) dfrA12	IncFII, IncI1-I(Alpha), IncFIB(K), p0111, IncX1	afaB afaC ecpA-E elfC elfD elfG hcpA-C papI fimD fimF fimG fimH aatA ibeB ibeC tia espL1 espL4 espR1 espX1 espX4 espX5 hlyE/clyA	3	310-104kb	~120	121,928	IncFII/IncX1
	W6-B6P1	Escherichia coli	562	4923834	142	102987	50.70%	3871	H5 O142	aph(3")-Ib aadA2 aph(6)-Id blaCTX-M-27 blaTEM-1B floR qnrS1 sul2 sul3 tet(X4) tet(A) dfrA12 dfrA14	IncFII, IncI1-I(Alpha), IncFIB(K), p0111, IncX1	afaB afaC ecpA-E elfC elfD elfG hcpA-C papI fimD fimF fimG fimH aatA ibeB ibeC tia espL1 espL4 espR1 espX1 espX4 espX5 hlyE/clyA	4	310-104kb	~130		
	W7-C6P1	Escherichia coli	562	4931041	144	104007	50.70%	3871	H5 O142	aph(3")-Ib aadA2 aph(6)-Id blaCTX-M-27 blaTEM-1B floR qnrS1 sul2 sul3 tet(X4) tet(A) dfrA12 dfrA14	IncFII IncI1-I(Alpha) p0111 IncFIB(K) IncX1	afaB afaC ecpA-E elfA elfC elfD elfG hcpA-C papI fimD fimF fimG fimH aatA ibeB ibeC tia espL1 espL4 espR1 espX1 espX4 espX5 hlyE/clyA	4	310-104kb	~120		
	W8-B1P1	Escherichia coli	562	4929203	146	104008	50.70%	3871	H5 O142	aph(3")-Ib aadA2 aph(6)-Id blaCTX-M-27 blaTEM-1B floR qnrS1 sul2 sul3 tet(X4) tet(A) dfrA12 dfrA14	IncFII IncI1-I(Alpha) p0111 IncFIB(K) IncX1	afaB afaC ecpA-E elfA elfC elfD elfG hcpA-C papI fimD fimF fimG fimH aatA ibeB ibeC tia espL1 espL4 espR1 espX1 espX4 espX5 hlyE/clyA	4	310-104kb	~150		
	W9-A4E1	Escherichia coli	562	4927392	147	102987	50.70%	3871	H5 O142	aph(3")-Ib aadA2 aph(6)-Id blaCTX-M-27 blaTEM-1B floR qnrS1 sul2 sul3 tet(X4) tet(A) dfrA12 dfrA14	IncFII IncI1-I(Alpha) p0111 IncFIB(K) IncX1	afaB afaC ecpA-E elfA elfC elfD elfG hcpA-C papI fimD fimF fimG fimH aatA ibeB ibeC tia espL1 espL4 espR1 espX1 espX4 espX5 hlyE/clyA	4	310kb-78kb	~130		
	W10-C6P2	Escherichia coli	562	4931298	140	104138	50.70%	3871	H5 O142	aph(3")-Ib aadA2 aph(6)-Id blaCTX-M-27 blaTEM-1B floR qnrS1 sul2 sul3 tet(X4) tet(A) dfrA12 dfrA14	IncFII IncI1-I(Alpha) p0111 IncFIB(K) IncX1	afaB afaC ecpA-E elfC elfD elfG hcpA-C papI fimD fimF fimG fimH aatA ibeB ibeC tia espL1 espL4 espR1 espX1 espX4 espX5 hlyE/clyA	3	310kb-115kb	~150		
Negative	W3-F2E1	Escherichia coli	562	5301513	117	106020	50.30%	641	O45:H21	aph(4)-Ia aac(3)-IV aadA1 aadA5 aac(6')-Ib3 blaNDM-5 blaTEM-1B blaOXA-10 blaCTX-M-14 mcr-1.11 fosA3 cmlA1 floR OqxB OqxA ARR-2 sul2 tet(A) dfrA14 dfrA17 bleO	IncHI2 IncFII IncHI2A IncFIB(AP001918) p0111	afaB afaC cfaA-E ecpA-E elfA elfC elfD elfG eaeH hcpA-C papA papC papD papH fimA fimC-I aatA ehaB upaG/ehaG ibeB ibeC tia espL1 espL4 espR1 espX1 espX4 espX5 aec31 aec32 hlyE/clyA					
	W12-F2E2	Escherichia coli	562	5301205	133	99484	50.30%	641	O45:H22	aac(3)-IId aph(6)-Id aadA5 aadA22 aph(3')-Ia mph(A) floR sul2 tet(M) tet(A) dfrA17	IncHI2 IncFII IncHI2A IncFIB(AP001918) p0111	afaB afaC cfaA-C ecpA-E elfA elfC elfD elfG eaeH hcpA-C papA papC papD papH fimA fimC-I aatA ehaB upaG/ehaG ibeB ibeC tia espL1 espL4 espR1 espX1 espX4 espX5 aec31 aec32 hlyE/clyA					
	M1G2	Escherichia coli	562	4987949	146	109879	50.60%	48	O15:H11	aac(3)-IId aph(6)-Id aadA5 aadA22 aph(3')-Ia mph(A) floR sul2 tet(M) tet(A) dfrA17	IncFII IncI1-I(Alpha) IncFIB(K) p0111 IncX1	ecpA-E elfA elfC elfD elfG eaeH hcpA-C papA papC papD fimA fimC-I aatA ehaB upaG/ehaG ibeB ibeC espL1 espL4 espR1 espX1 espX4 espX5 espY1 aec15-22 aec25-32 hlyE/clyA					

Supplementary Table 1: Bioinformatics analysis and *tet*(X)-positive plasmid location.

Antimicrobial susceptibility testing													
		MIC values (mg/L)											
		tet(X)-positive strains									tet(X)-negative strains		
		W1-A6P1	W2-B6P2	W4-B1P2	W5-C5E1	W6-B6P1	W7-C6P1	W8-B1P1	W9-A4E1	W10-C6P2	W3-F2E1	W12-F2E2	M1G2
Meropenem	MEM	＜2	＜2	＜2	＜2	＜2	＜2	＜2	＜2	＜2	＜2	＜2	64
Aztreonam	ATM	32	16	16	64	32	8	64	16	16	＜2	＜2	8
Ampicillin	AMP	2048	2048	2048	2048	2048	2048	2048	256	256	＜2	8	2048
Ceftazidime	CAZ	32	16	16	64	16	16	16	16	16	＜2	＜2	2048
Cefepime	FEP	512	256	128	512	512	256	256	32	128	＜2	＜2	512
Gentamicin	GEN	＜2	＜2	＜2	＜2	＜2	＜2	＜2	＜2	＜2	512	256	128
Chloramphenicol	CHL	128	128	128	128	128	128	128	256	128	512	512	128
Polymyxin	PMX	＜2	＜2	＜2	＜2	＜2	＜2	＜2	＜2	＜2	＜2	＜2	＜2
Kanamycin	KAN	4	4	4	4	4	8	4	8	4	1024	512	512
Fosfomycin	FOS	128	16	128	1024	64	256	1024	64	128	512	256	2048
Ciprofloxacin	CIP	＜2	＜2	＜2	＜2	＜2	＜2	＜2	＜2	＜2	＜2	＜2	4
Sulfamethoxazole	SXT	2048	2048	2048	2048	2048	2048	2048	512	1024	2048	256	2048
Azithromycin	AZM	＜2	＜2	＜2	＜2	＜2	＜2	＜2	＜2	＜2	128	64	＜2
Tetracycline	TET	512	256	512	512	512	256	512	512	512	64	64	128
Doxycycline	DOX	64	32	64	32	64	32	64	64	64	16	16	32
Tigecycline	TGC	16	8	8	16	32	8	16	16	16	0	＜2	＜2
Rifampin	RIF	8	8	8	4	4	4	4	2	2	8	8	128
Omadacycline	OMC	64	128	64	64	64	64	64	64	32	32	32	16
Transconjugant AST													
J53	TGC	16	8	8	8	16	8	16	32	16			
LGJ2		8	8	8	8	16	8	16	8	16			

Supplementary Table 2: The MICs for *tet*(X)-positive and -negative strains.

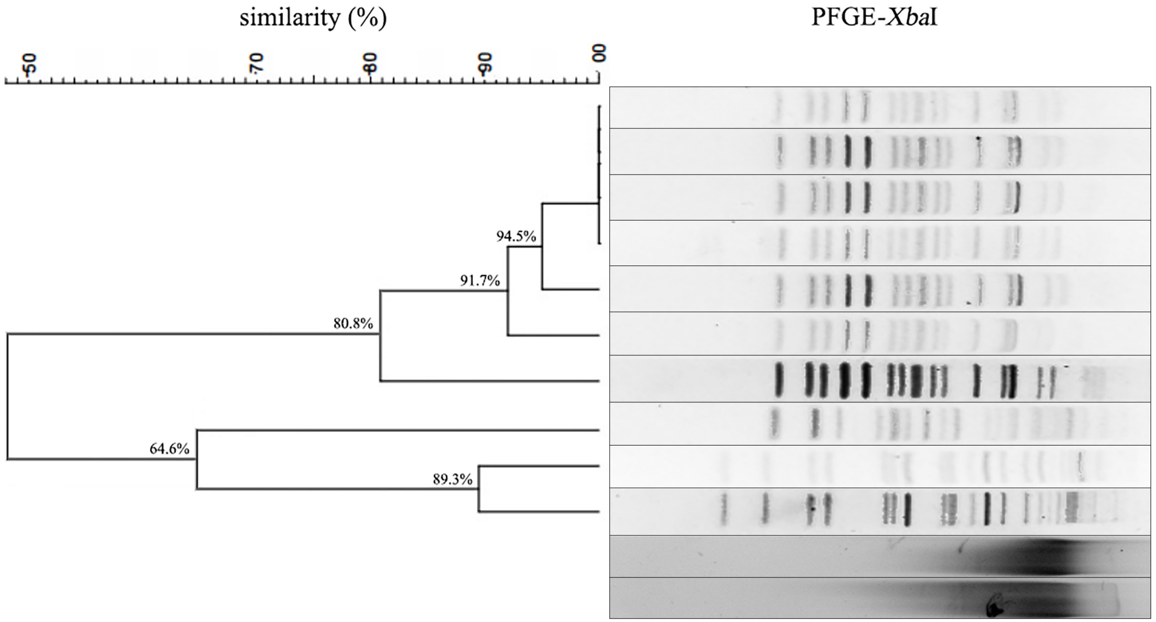

isolate	Group	MLST
W1	A	ST3871
W4	A	ST3871
W5	A	ST3871
W7	A	ST3871
W8	A	ST3871
W9	A	ST3871
W10	A	ST3871
M1G2	B	ST48
W3	C	ST641
W12	C	ST641
W6		ST3871
W11		ST3871

Fig. S1 A XbaI-digested PFGE dendrogram containing clear and unclear bands.

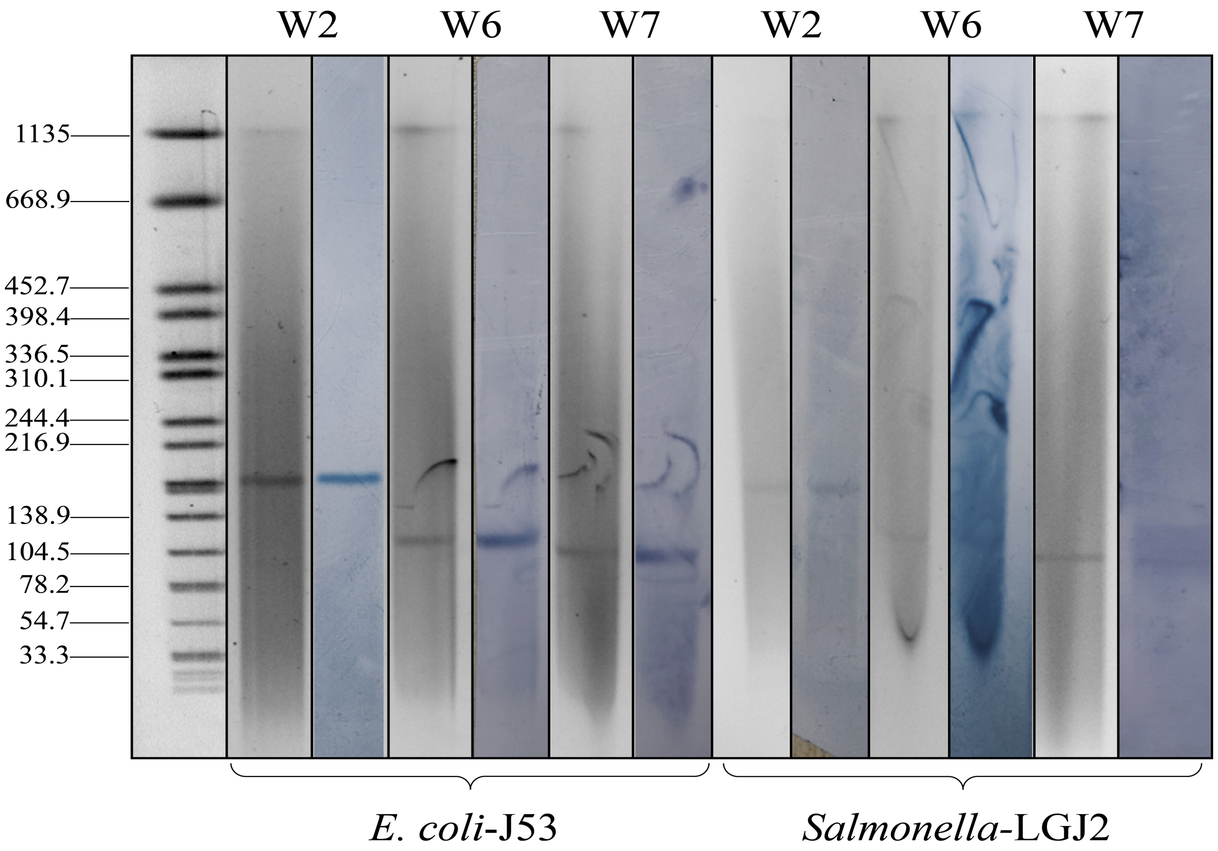

Fig. S2 The *tet*(X)-positive plasmid location in recipient strains using S1-PFGE and Southern blotting.
